# Remarkable Case of Catalepsy

**Published:** 1853-08

**Authors:** 


*Remarkable case of Catalepsy.*—Cornelius Vroomer, now aged about 37 years, a country laborer, and resident of Clarkson, Monroe Co., N. Y., of robust and vigorous frame, middle size, and 165 lbs. weight, about five years since (in June, 1848) made complaint of an obtuse pain in his head and epigastrium. This was sufficient to induce him to stop work, and ask medical advice. Dr. C., of Clarkson, saw him and prescribed little more than guaiacum and rhubarb, supposing the case one that would prove amenable to mild measures. The pain in the head and stomach continued for about two weeks, when conciousness, sensorial powers and volition became suspended. His respiration and circulation continued natural, while, without coma or spasm, muscular rigidity retained the body and limbs in the precise position they were when the attack came on. No apparent change occurred from this condition for several days, and with the exception of waking periods of from one to sixteen hours taking place at intervals of from one week to four months, he has remained in the same condition to this date.

His conscious periods for the first few months occcurred at intervals seldom exceeding two weeks—latterly seldom less than four, and sometimes not less than eight or ten weeks. His most lengthy period of unconsciousness has been four months; longest waking interval sixteen hours. In the great length of the periods of unconsciousness and motionless rigidity, the case is unique and partioularly interesting. During the whole of these periods there has been the most complete anæsthesia. Powerful excitants have been applied to the surface at various times, such as blisters over the head and spine, and at one time, accidentally, sufficient heat to his limbs to vesicate extensively, without causing any manifestation of pain. The nervous system, it would appear, is but slightly influenced by all external impressions. None but the slightest motion of the limbs are induced by current of magnetic electricity, and under repeated application of cold water, including a plunge into Lake Ontario, he seemed to experience no impression, excepting at one time under a long-continued douche from a height, he moved himself from his position, but did not manifest a full return of sense.

The involuntary functions of his system are performed with much regularity, but with less force and activity than in health. Pulse and respiration regular but faint. Evacuation from his bowels occurs once in from six to nine days—passes his urine more frequently. These calls of nature are made manifest by a slight uneasiness of his body, and are kept in abeyance until necessary preparations are made. Erom this feature of the case, and the fact that when conscious he has once or twice evinced a knowledge of events which transpired during his cataleptic state, and that he possesses the power of balancing himself when placed erect upon his feet, it is apparent to the physiologist that everything but the spinal and ganglionic system is not asleep, that the senses are partly awake, and of a somnambulic character. I am informed by his attendants that there are times when his system seems more completely under the influence of natural sleep than at others; this soporose state is indicated by snoring, and is not attended with the least relaxation of the muscular rigidity. Food is usually administered to him twice a-day, and oniy by prying the jaws asunder and forcibly introducing it into his mouth and fauces.

His conscious periods return suddenly and at irregular intervals. During these short waking periods he walks erect, shows no very manifest diminution of strength or natural mobility, greets his old acquaintances, converses freely, and supplies liberally the demands of his stomach. On one occasion, not many weeks since, it is said he walked to the beach of the Lake, a distance of half a mile, and treated himself and friends to a supply of such grocery luxuries as dried herring and crackers.

Nothing is known of his habits previous to the invasion of this disease, to which it can be referred. It is known that he "imbibed" only occasionally, and was a tobacco chewer, and it is worthy of note that the last-named habit is completely reformed, as he has not expressed a desire for the weed in four years. During the colder months the temperature of his surface is considerably diminished, and at time his extremities are quite cold. When the surrounding atmosphere is comfortably warm, the temperature varies but little, if any, from the healthy standard.

As seen at this time his cast of features is calm and complacent, and presents nothing particularly unpleasing, except the protrusion of his lips. The firm pressure of his jaws has forced the incisor teeth to a very acute angle in front, and almost forced them from their sockets. The projection of the teeth and the approximation of the maxillæ have forced his teeth to a prominence quite equal to his nose. The external surface of the upper lip lies in close proximity with the nostrils.

Tonic spasm, of not the greatest rigidity, exists throughout his muscular system. His head is brought downward and forward towards the sternum; the body is curved forward, the spine forming the arc of a circle, as in emprosthotonos. The forearms cross the abdomen; the wrist joints and those of the fingers are the most firmly fixed. The thighs and knees are but slightly flexed, and are firmly fixed at obtuse angles. When placed upon his feet or seated in a chair, he has the power of sustaining himself in an erect posture as rigid as a statue, from which position he has to be removed mechanically by his attendants. At one time he was placed upright upon his feet, and suffered to remain in his motionless position for three days and nights.

It is unecessary to specify the various measures which have been adopted by a number of the sons of Æsculapius for his restoration. Their "science and skill," thus far, have seemed to avail nothing in this case. His friends say they have restored consciousness on one or two occasions by administering very copious draughts of whiskey. Nitrous oxide, long continued cold to the head and spine, and chloroform, are measures which have not been tested.—*N. Y. Democrat.*

